# Advances in research on pharmacological mechanisms of anatabine: from nicotinic modulation to multitarget therapeutic potential

**DOI:** 10.3389/ebm.2026.11069

**Published:** 2026-06-29

**Authors:** Xiaonan Li, Xiaomin Liu, Huaquan Sheng, Jianfeng Guo, Leihao Zhang, Ting Fei, Yihan Gao

**Affiliations:** Basic Research Department, Shanghai New Tobacco Product Research Institute Co., Ltd. (SNTPRI), Shanghai, China

**Keywords:** alkaloids, Alzheimer’s disease, anatabine, anti-inflammatory, neurological disorders, NF-κB, pharmacological mechanisms

## Abstract

Anatabine, a characteristic minor alkaloid derived from tobacco byproducts, exhibits unique structural analogy to nicotine but possesses a superior safety profile and lower addictive liability, rendering it a promising natural multi-target therapeutic candidate. Accumulating preclinical evidence has demonstrated that anatabine exerts neuroprotective, anti-inflammatory, and antioxidant effects mainly through modulating α7/α4β2 nicotinic acetylcholine receptors, suppressing NF-κB/STAT3 inflammatory signaling, and activating the Nrf2-mediated antioxidant pathway. It effectively ameliorates typical pathological alterations, including β-amyloid deposition, tau hyperphosphorylation, and microglial overactivation, thereby improving cognitive and behavioral deficits in neurodegenerative disease models. Additionally, anatabine displays broad pharmacological potentials in chronic inflammation, autoimmune thyroiditis, asthma, and hypertension. Differing from previous reviews that merely focused on single receptor regulation, the present work systematically summarizes the multi-target pharmacological characteristics of anatabine, comprehensively collates its preclinical efficacy across multiple disease categories, and highlights its advantages over nicotine in safety and addiction risk. Furthermore, we analyze the current limitations, druggability optimization challenges, and clinical translation prospects, and propose sustainable strategies for high-value utilization of tobacco byproducts. This review provides an updated and systematic theoretical basis for further mechanism exploration and therapeutic development of anatabine.

## Impact statement

This manuscript provides a comprehensive and systematic synthesis of the rapidly expanding body of evidence on anatabine, a minor tobacco alkaloid, positioning it as a promising multi-functional therapeutic agent. Moving beyond its established role as a partial nicotinic receptor agonist, we delineate its novel multi-target mechanisms of action, including the synergistic suppression of key inflammatory pathways (NF-κB, STAT3) and activation of the Nrf2-mediated antioxidant response. We critically evaluate its efficacy across a wide spectrum of preclinical models, including: 1) Neurodegenerative disorders (Alzheimer's disease, chronic traumatic encephalopathy); 2) Chronic inflammatory conditions (ulcerative colitis, asthma, rosacea); 3) Autoimmune and endocrine dysfunctions (Hashimoto's thyroiditis); 4) Cardiovascular disease (hypertension).

## Introduction

Tobacco contains abundant specialized metabolites, among which alkaloids are the most pharmacologically active constituents [1, 2]. Nicotine has long been the research focus of tobacco alkaloids, while a large number of minor alkaloids from tobacco by-products remain underutilized and lack in-depth pharmacological exploration. Massive discarded tobacco leaves are produced during planting and industrial processing, which are rich in trace bioactive alkaloids and represent an important resource for natural drug discovery and high-value reuse of agricultural by-products [3–5]. Nevertheless, anatabine, a therapeutically valuable minor tobacco alkaloid, has not yet received systematic pharmacological characterization and developmental exploitation, resulting in a clear research gap in its mechanism elucidation and translational application.

Nicotinic acetylcholine receptors (nAChRs) are ligand-gated ion channels widely distributed in the central and peripheral nervous systems [6]. They play critical roles in regulating neuronal excitability, neurotransmitter release, inflammation and immune function [7–10]. As the primary molecular targets of nicotine and most tobacco alkaloids, different nAChR subtypes exhibit distinct tissue distribution and pharmacological properties, which underlie the diverse biological activities of alkaloid compounds [11]. Nevertheless, relevant studies investigating binding interactions between minor alkaloids and nAChRs remain scarce [12, 13]. Existing research has largely centered on nicotine, while the binding affinity, subtype selectivity and subsequent functional impacts of most trace tobacco alkaloids at nAChRs lack systematic characterization, hindering full elucidation of their *in vivo* pharmacological mechanisms [14].

**FIGURE 1:**
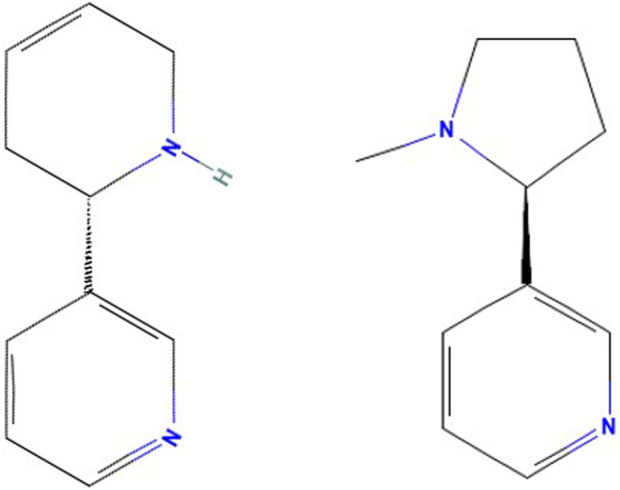
Structural comparison of chemical skeletons between anatabine and nicotine. Anatabine (left) contains a six-membered piperidine nitrogen heterocycle; its pyridine ring is attached to the carbon atom adjacent to the piperidine nitrogen, forming a secondary amine with an N–H bond. Nicotine (right) consists of a five-membered pyrrolidine saturated nitrogen heterocycle, with its pyridine ring bound to the carbon atom next to the pyrrolidine nitrogen and adopting a tertiary amine structure that lacks an N–H bond. This structural difference fundamentally accounts for their distinct receptor affinity, safety profiles, and addictive potential.

Anatabine is a characteristic minor alkaloid mainly enriched in Nicotiana plant roots [15, 16]. It shares high structural homology with nicotine but possesses a better safety profile and markedly lower addictive risk [17] (Figure 1). Mechanistically, anatabine acts as a functional modulator of α7/α4β2 nicotinic acetylcholine receptor subtypes. Beyond nAChR regulation, it exerts multi-target pharmacological effects via suppressing NF-κB/STAT3 inflammatory cascades and activating Nrf2-mediated antioxidant signaling. Mounting preclinical evidence has validated its neuroprotective, anti-inflammatory, immunomodulatory and anti-oxidative activities, with promising therapeutic potential covering neurodegenerative diseases, chronic inflammatory disorders, autoimmune endocrine diseases and hypertension.

Nicotine exhibits definite neuroprotective and anti-inflammatory properties, yet its inherent addictive liability and potential neurotoxicity greatly limit clinical transformation and long-term medication application [18–21]. As a structural analog of nicotine with superior safety advantages, anatabine has become a promising alternative natural candidate [22]. However, existing studies are fragmented: there lacks a comprehensive overview of its multitarget pharmacological mechanisms, a systematic collation of preclinical efficacy across multiple disease models, and an in-depth summary of current bottlenecks and future optimization directions for clinical translation [23–25].

This review systematically consolidates the latest research advances of anatabine, focusing on its paradigm shift from single nicotinic receptor modulation to multi-target regulation of inflammation and oxidative stress. We critically summarize its pharmacological characteristics and preclinical efficacy in neurodegeneration, chronic inflammation, endocrine dysfunction and cardiovascular diseases, compare its superiority with nicotine in safety and dependence risk, and further highlight current limitations, druggability optimization strategies and clinical translation prospects. This work aims to provide theoretical reference for the development of anatabine-based natural therapeutics and realize the sustainable high-value utilization of tobacco by-products.

## Literature search and methodology

Literature retrieval was systematically performed across PubMed, Web of Science, ScienceDirect, Google Scholar, and CNKI databases. The main search keywords included anatabine, tobacco minor alkaloid, nicotinic acetylcholine receptor, neuroprotection, Alzheimer’s disease, inflammation, NF-κB, Nrf2. All eligible literature published by the year 2025 was retrieved for analysis. Original research articles, review papers, and clinical studies published in English or Chinese were included. Exclusion criteria were as follows: conference abstracts, letters, duplicate publications, non-peer-reviewed gray literature, and studies with incomplete experimental data or ambiguous results. All retrieved literatures were further screened by title, abstract and full text to finally collect eligible references for this review.

## Regulation of *β*-amyloid plaques and tau phosphorylation in Alzheimer’s disease models

Alzheimer’s Disease (AD) is a neurodegenerative disorder characterized primarily by progressive cognitive dysfunction and memory decline [26, 27]. The key pathological hallmark is characterized by the widespread deposition of β-amyloid (Aβ) plaques derived from proteolytic cleavage of the β-amyloid precursor protein (β-APP) within the neocortical and hippocampal architectures [28–30]. β-APP is a transmembrane glycoprotein predominantly expressed in neuronal membranes, and its processing generates Aβ peptides, with Aβ_1-40_ and Aβ_1-42_ are the most prevalent isoforms with distinct aggregation kinetics and neurotoxicity profiles [31–33]. In transgenic AD mouse models, D. Paris et al. demonstrated that intraperitoneal administration of anatabine significantly reduced cerebral Aβ levels. Pharmacokinetic profiling indicated substantially higher brain exposure than plasma exposure (Brain Cmax: 5194.4 ± 339.3 ng/mL vs. Plasma Cmax: 1,501.7 ± 138.6 ng/mL). Critically, anatabine treatment reduced cerebral Aβ_1–40_ and Aβ_1–42_ levels by 30%–40% (p < 0.05) compared with PBS-treated controls. *In vivo* studies employing Tg-PS1-APPswe transgenic AD murine models elucidated anatabine’s therapeutic potential through comprehensive behavioral assessments and cerebral disease-associated protein profiling. Verma M et al. further confirmed that oral anatabine ameliorated dementia-like behavioral deficits while reducing microglial activation and plaque burden [34]. *In vitro* studies using 7W-CHO cells overexpressing human APP revealed that anatabine administration during cell culture significantly suppressed Aβ_1-40_ and Aβ_1-42_ plaque formation, demonstrating dose-dependent efficacy (p < 0.001), with particularly potent suppression of the Aβ_1-40_ isoform compared to Aβ_1-42_ [35]. Further mechanistic analysis confirmed that anatabine robustly suppresses the expression of phosphorylated p65 NF-κB and TNFα (p < 0.001). As NF-κB is a central regulator of neuroinflammation and modulates the expression of BACE-1, the rate-limiting enzyme in Aβ generation [36–41]. Consistent with this, anatabine significantly reduced BACE-1 expression in SH-SY5Y neuronal cells (p < 0.05), supporting its relevance as a therapeutic target in AD [42]. These findings highlight anatabine as a promising candidate worthy of further investigation for the development of novel therapeutic interventions against AD.

While the precise pathogenesis of AD remains elusive, current clinical management predominantly relies on acetylcholinesterase inhibitors (AChEIs). Notably, therapeutic exploration of anatabine remains limited, notwithstanding its demonstrated neuroprotective properties in preclinical models through multitarget mechanisms involving α7-nAChR modulation and NLRP3 inflammasome suppression [43, 44]. Furthermore, radioligand binding assays validated the differential binding affinities of anatabine and its stereoisomers towards α4β2/α7 nAChR subtypes. Both S- and R-enantiomers demonstrate potent agonistic activity at α7 nAChRs (EC_50_ = 69.7 ± 30 μM and 51.8 ± 6.5 μM respectively), with concomitant partial agonist efficacy at α4β2 nAChRs (EC_50_ = 2.64 ± 1.4 μM and 0.74 ± 0.21 μM respectively) [45, 46]. This investigation substantiates anatabine’s stereochemical configuration-dependent therapeutic potential as a multimodal neuromodulator, particularly for adjunctive management of neuropsychiatric disorders through coordinated α7/α4β2 receptor activation paradigms.

Beyond Aβ pathology, tau protein abnormalities are another defining feature of AD and related tauopathies [47, 48]. Under physiological conditions, tau stabilizes microtubule networks through dynamic interactions with tubulin dimers. Pathological hyperphosphorylation or aberrant folding of tau induces intracellular neurofibrillary tangles (NFTs), culminating in axonal transport disruption and neuronal dysfunction [49]. Targeting tau phosphorylation is therefore a rational therapeutic strategy [50–52]. In tauopathy transgenic murine models, oral administration of Anatabine ameliorated hindlimb clasping phenotypes and mitigated transgene-induced paralysis. Molecular analyses demonstrated significant reductions in p-tau epitopes in brain and spinal cord tissues, particularly at PHF-1 (pSer396/404), RZ3 (pThr231), and CP13 (pSer202) sites [53]. Thus, Anatabine demonstrates therapeutic efficacy in tauopathy models by ameliorating behavioral deficits and concurrently reducing pathological tau phosphorylation at key epitopes in the central nervous system.

## Specific modulation of cognitive, motor, and memory deficits induced by antagonists and inhibitors

Previous research on nicotine has indicated that its agonistic action on central cholinergic receptors can acutely enhance several cognitive domains, including sensory processing, attentional performance, information integration, and motor responsiveness [54–56]. Anatabine, which shares structural similarity with nicotine, has also been validated by multiple studies for its regulatory effects on certain cognitive functions [57]. Moreover, whereas nicotine exhibits pronounced effects on anxiety-related emotional responses, anatabine has also demonstrated measurable anxiolytic efficacy in preclinical behavioral models [58].

Chronic Traumatic Encephalopathy (CTE) is a progressive neurodegenerative disease associated with repetitive head trauma in patients. An initial symptom in CTE patients is short-term memory loss, which guradually progresses to cognitive impairment and dementia. A pathological hallmark of CTE is the accumulation of hyperphosphorylated tau in neurons and glial cells, although its spatial and temporal distribution differs significantly from that in AD [59, 60]. Morin, A. et al. treated mice subjected to two distinct injury models (5r-mTBI: 5 hits over 9 days/24r-mTBI: 24 hits over 90 days) with Anatabine (20 mg/kg/day for 90 days). Compared to the vehicle group, Anatabine treatment significantly inhibited astroglial hyperplasia in both injury models (p < 0.01) and ameliorated spatial memory deficits in the 24r-mTBI group. Additionally, Anatabine reduced the phosphorylation levels of Tau and NF-κB in the brain-injured animals [61]. Collectively, these findings demonstrate Anatabine’s potential to mitigate key pathological features of experimental CTE, including gliosis, tau hyperphosphorylation, neuroinflammation (via NF-κB), and associated cognitive deficits.

In another study, Levin et al. induced attentional dysfunction in Sprague–Dawley rats using the NMDA receptor antagonist dizocilpine (MK-801). Short-term anatabine treatment significantly alleviated the resulting attention deficits, suggesting a potential facilitatory role in memory and attentional performance [62]. Additional work has examined the combined effects of nicotine and anatabine on motor behavior. Clemens et al. administered intravenous nicotine along with minor tobacco alkaloids, including anatabine, and observed enhanced general locomotor activity relative to nicotine alone [63]. Patrick M. Callahan et al., utilizing the classic Y-maze and novel object recognition (NOR) paradigms to assess memory, found that anatabine inhibited scopolamine-induced spatial memory deficits. Anatabine demonstrated a unique profile in modulating short-term spatial memory [64]. Wiley, J.L. et al. directly compared the effects of nicotine and anatabine on locomotor behavior in SD rats. A low dose of anatabine (1 mg/kg) markedly increased locomotor activity, while higher doses of anatabine resulted in a decrease in locomotion [65]. Together, these findings show that anatabine can alleviate attention deficits, modulate locomotor activity in a dose-dependent manner, and protect against spatial memory impairment. These combined neuromodulatory effects highlight its broad therapeutic potential in neurocognitive disorders.

## Therapeutic attenuation of diverse chronic inflammatory conditions

Inflammation is a pathological response triggered by biological, chemical, or physical stimuli. Chronic inflammation, in contrast, reflect a sustained and dysregulated immune response [66, 67]. Pro-inflammatory cytokines (e.g., TNFα, IL-6) play pivotal roles in the development of chronic inflammatory diseases through signaling pathways such as p38 MAPK and IL-6/JAK/STAT3 [68, 69]. Accordingly, monitoring established inflammartory biomarkers helps to evaluate disease severity [70, 71]. As an over-the-counter dietary supplement, the anti-inflammatory effects of Anatabine have been supported by clinical evidence [72]. Recent research continues to elucidate its therapeutic potential across multiple inflammatory conditions.

### Ulcerative colitis

A three-dimensional *in vitro* intestinal inflammation model (Caco-2/HT29-MTX/THP-1 co-culture) demonstrated that anatabine improved epithelial integrity and reduced inflammatory cytokine release, as indicated by increased transmembrane electrical resistance (TEER) values and decreased permeability [73]. In murine models of colitis, oral anatabine administration reduced pro-inflammatory cytokine levels while increasing anti-inflammatory cytokine interleukin-10 (IL-10) expression [74, 75]. However, in dextran sulfate sodium (DSS)-induced ulcerative colitis (UC) models, the anti-inflammatory efficacy of anatabine was generally weaker than that of nicotine [76]. Similarly, other scholars induced colitis in male Wistar rats using DSS and administered anatabine via intraperitoneal injection at a dosage equivalent to that of nicotine. The therapeutic outcomes for UC were markedly different between anatabine and nicotine treatments; specifically, anatabine produced weaker anti-inflammatory effects, with a milder reduction of pro-inflammatory cytokines and less obvious improvement of intestinal epithelial barrier injury compared with nicotine [77]. These cumulative findings substantiate a nuanced role for anatabine in mitigating intestinal inflammation, yet they consistently highlight a divergent efficacy profile compared to nicotine. This compelling disparity necessitates a more rigorous dissection of its mechanistic underpinnings and therapeutic potential through integrated *in vitro* and *in vivo* studies to firmly establish its translational value.

### Chronic neuroinflammation

In the study by Paris D et al., anatabine was found to inhibit the inflammatory response by modulating the phosphorylation status of STAT3, consequently regulating the expression of TNF-α and interleukin-6 (IL-6) [78]. Notably, this specific mechanism is also implicated in the pathogenesis of Alzheimer’s disease (AD). Chronic neuroinflammation is a common feature in the progression of AD. Beyond its documented ability to significantly suppress β-amyloid expression, anatabine further mitigates neuroinflammation by regulating the activation states of both STAT3 and NF-κB, leading to a downstream reduction in the expression levels of key target genes such as Bace1, iNOS, and Cox-2 [34, 79]. These findings support its therapeutic potential in mitigating neuroinflammatory cascades associated with neurodegeneration.

### Rosacea

Rosacea is a common chronic inflammatory skin disorder [80], in which dysregulated inflammatory signaling plays a key pathogenic role [81, 82]. Lanier R.K. et al. developed and evaluated a topical ointment containing Anatabine. Following 30 days of application by ten rosacea patients, significant improvement in rosacea symptoms was observed, with no reported complications or adverse effects, indicating good tolerability. This study on rosacea proposed that the anti-inflammatory effects of Anatabine upon topical application may arise from its inhibition of NF-κB activation or the suppression of other related pro-inflammatory signaling transduction mechanisms [83]. These data validate the potential of topical anatabine as a promising therapeutic candidate for rosacea, demonstrating targeted anti-inflammatory efficacy and a favorable safety profile. Nevertheless, future studies specifically evaluating cutaneous sensitivity and long-term tolerability are warranted to strengthen the clinical evidence base.

### Chronic joint pain

Chronic joint pain is commonly treated with intra-articular hyaluronic acid or NSAIDs [84]. However, recent studies have found that anatabine, as a dietary supplement, exhibits certain alleviating effects on symptoms such as joint pain and stiffness [72]. Meanwhile, some research suggests a negative correlation between tobacco product use and the incidence of knee osteoarthritis, though more scientific and systematic statistical analysis is still required [85] Other studies indicate that anatabine does not affect the level of the inflammatory marker TNF-α in regulating local muscle damage caused by exercise, implying that its anti-inflammatory effects may vary depending on the type of inflammation as well as patient-specific factors such as age [86, 87]. Overall, these findings confirm the potential role of anatabine in joint health management, while also highlighting the need for further mechanistic and clinical investigations to clarify its context-dependent efficacy.

### Hypertension

Epidemiological and metabolomic studies have revealed a close association between endogenous anatabine level and hypertension. Compared with normotensive Wistar-Kyoto rats, spontaneously hypertensive rats showed significantly lower anatabine concentrations in feces, blood and hypothalamic paraventricular nucleus (PVN), implying that anatabine deficiency may contribute to hypertensive pathogenesis. Long-term subcutaneous infusion of anatabine could effectively lower blood pressure in hypertensive models. Mechanistically, anatabine inhibits NF-κB activation in PVN microglia, thereby suppressing NLRP3 inflammasome-mediated pyroptosis and reducing the release of pro-inflammatory factors and oxidative stress. Such central regulation ultimately decreases sympathetic nervous system overactivity and exerts a stable antihypertensive effect. Collectively, anatabine represents a promising natural candidate for hypertension intervention via targeting central neuroinflammatory and oxidative signaling pathways.

Recent studies suggest that hypertension is associated with activated inflammatory responses, and numerous studies indicate that chronic inflammation accompanies the onset and progression of hypertension [88, 89]. A 2025 study revealed that spontaneously hypertensive rats (SHRs) exhibited significantly lower levels of anatabine in feces, blood, and the paraventricular nucleus (PVN) compared to normotensive Wistar-Kyoto rats (WKY). Metabolomic analysis suggested that anatabine deficiency may be linked to the pathogenesis of hypertension. After 12 weeks of continuous subcutaneous infusion of anatabine solution (0.014 mg/kg/min, approximately equivalent to 20.16 mg/kg/day), treatment with anatabine was found to inhibit NF-κB activation in microglia within the PVN, thereby suppressing NLRP3 inflammasome activation and Caspase-1-dependent pyroptosis. This led to reduced release of inflammatory factors and oxidative stress, ultimately decreasing sympathetic nervous activity and blood pressure [90]. This study elucidates that anatabine, as a natural alkaloid, may confer antihypertensive effects via central nervous mechanisms, offering a novel potential target and therapeutic strategy for hypertension treatment.

### Asthma

Asthma is a chronic inflammatory airway disease [91]. Abdo W. et al. demonstrated that anatabine alleviates allergic asthma through a dual-pathway mechanism involving the activation of the Nrf2/HO-1 pathway and synergistic suppression of NF-κB signaling [92]. Among these, the Nrf2/HO-1 axis has been established as a potential therapeutic target for asthma treatment [93]. In addition, Messinis, D. E. et al. utilized four cell lines (HEK-293, SH-SY5Y, PMA-differentiated THP-1, and human primary epidermal keratinocytes) to validate that anatabine inhibits dual-specificity phosphatases (DUSPs), thereby activating MAPK (p38/JNK/ERK) signaling and promoting NRF2 nuclear translocation. This leads to the upregulation of antioxidant genes (e.g., HMOX1, NQO1), enhancing cellular antioxidant capacity, and concurrently suppressing the NF-κB/STAT3 inflammatory pathway [94]. Using a systems biology approach, this study revealed for the first time that anatabine serves as a potent activator of NRF2. Together, these findings provide preclinical evidence supporting anatabine as a potential natural therapeutic agent against asthma.

### Thyroiditis

Additional studies have indicated that anatabine may also possess certain therapeutic effects against thyroiditis. For instance, research by Schmeltz L.R. et al. demonstrated that patients with Hashimoto’s thyroiditis who received anatabine supplementation for 3 months exhibited a significant reduction in TgAb levels, although TPOAb levels remained unaffected [95]. It is hypothesized that anatabine functions as an α4β2/α7 nicotinic acetylcholine receptor agonist during the treatment of thyroiditis, thereby inhibiting the production of interleukin-1β (IL-1β) and interleukin-18 (IL-18) [96–100]. These findings indicate a potential role for anatabine in autoimmune thyroid disease modulation.

## Discussion and future perspectives

This review systematically summarizes the multitarget pharmacological properties and preclinical therapeutic evidence of anatabine, a representative minor tobacco alkaloid. Distinct from nicotine, anatabine shares high structural homology while possessing a more favorable safety profile and markedly lower addictive potential. It modulates α7/α4β2 nAChRs and acts as a key upstream regulator of the NF-κB/STAT3 inflammatory axis and Nrf2 antioxidant signaling pathway, thereby intervening in neuroinflammation, oxidative stress, protein pathological deposition and immune dysfunction. Accumulated preclinical data confirm that anatabine alleviates Aβ plaque deposition, tau hyperphosphorylation, microglial overactivation and glial proliferation. It exhibits promising efficacy against Alzheimer’s disease, tauopathy, chronic traumatic encephalopathy, as well as multiple chronic inflammatory disorders including ulcerative colitis, rosacea, asthma, hypertension and autoimmune thyroiditis. Table 1 is provided as supplementary material due to space limitations.

**TABLE 1:** Summary of pharmacological mechanisms and effects of anatabine is provided in the supplementary attachment.

Disease category	Experimental model	Administration route	Key effects and mechanisms	Evidence strength	Consistency of findings	Main limitations	Ref.
Alzheimer’s disease	Tg-PS1-APPswe transgenic mice	Oral administration	Reduced cerebral An_1–40_ and Ad_1–42_ levels; attenuated microglial hyperplasia and dementia-like symptoms	Preclinical animal	Consistent	Mostly single transgenic model; lack of long-term survival and cognitive follow-up	[34]
	Overexpressing 7W-CHO cells	*In vitro* treatment	Dose-dependently suppressed Aβ plaque formation	*In vitro* cell	Consistent	Only cell-level verification; no further *in vivo* mechanism validation	[35]
	Human neuronal SH-SY5Y cells	*In vitro* treatment	Reduced BACE-1 expression; inhibited NF- mationrm survival and	*In vitro* cell	Consistent	Absence of primary neuronal validation	
Tauopathy (Tg tau P301S)	Tau transgenic mice	Oral administration	Ameliorated hindlimb clasping and paralysis; reduced tau hyperphosphorylation at key epitopes	Preclinical animal	Consistent	Limited behavioral indicators; few stereoisomer comparative studies	[53]
Chronic traumatic encephalopathy (CTE)	mTBI mouse models	Oral administration	Inhibited astroglial hyperplasia; improved spatial memory deficits	Preclinical animal	Consistent	Only rodent models; no large animal or clinical evidence	[61]
Attention & motor function deficit	SD rats	Subcutaneous injection	Alleviated attention deficits; regulated locomotor activity in a dose-dependent manner	Preclinical animal	Partially inconsistent	Efficacy varies with dose and modeling method; lack of unified dose standard	[62–64]
Ulcerative colitis	DSS-induced murine colitis	Oral administration	Alleviated intestinal inflammatory injury and pro-inflammatory cytokine release	Preclinical animal	Partially inconsistent	Efficacy weaker than nicotine in some studies; inconsistent modeling protocols	[74–77]
	3D Caco-2/HT29-MTX/THP-1 intestinal model	*In vitro* treatment	Improved epithelial barrier integrity and reduced inflammatory permeability	*In vitro* 3D model	Consistent	*In vitro* model cannot fully simulate *in vivo* intestinal microenvironment	[73]
Rosacea	Human patients	Topical ointment	Markedly improved clinical symptoms with good tolerability; potential inhibition of NF-itopesry a	Preliminary clinical	Consistent	Small sample size; open-label without placebo control	[83]
Hypertension	Spontaneously hypertensive rats (SHR)	Subcutaneous injection	Inhibited microglial NF-ve rats (SHR) placebo controlility; potential inhibition of N	Preclinical animal	Consistent	Only SHR strain; lack of validation in other hypertensive models	[90]
Asthma	Ovalbumin-induced asthmatic rats	Oral administration	Activated Nrf2/HO-1 antioxidant pathway; suppressed NF-κB inflammatory signaling	Preclinical animal	Consistent	Single induction model; no dose pathway; suppressed NF-κB inflamm	[92]
	HEK-293, SH-SY5Y and epidermal keratinocytes	*In vitro* treatment	Inhibited DUSPs; promoted NRF2 nuclear translocation and suppressed NF-κB/STAT3	*In vitro* cell	Consistent	Lack of primary airway cell verification	[94]
Hashimoto’s thyroiditis	Human patients	Oral supplementation	Significantly decreased TgAb levels; no obvious change in TPOAb	Preliminary clinical	Partially inconsistent	Limited clinical sample; unclear long-term intervention effect	[95]

**FIGURE 2:**
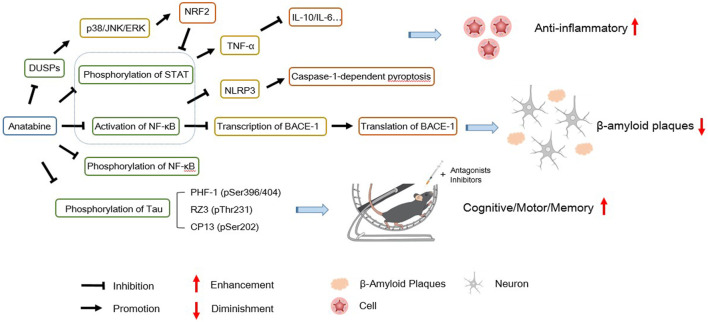
Schematic illustration of the multi-target pharmacological mechanism and therapeutic network of anatabine.

As illustrated in Figure 2, anatabine exerts its pharmacological effects mainly by activating α7/α4β2 nicotinic acetylcholine receptors, inhibiting the phosphorylation and activation of NF-κB and STAT3, and upregulating Nrf2-mediated antioxidant responses. It further downregulates downstream inflammatory mediators such as TNF-α, IL-6 and IL-1β, suppresses BACE1 expression, reduces β-amyloid deposition and relieves tau hyperphosphorylation at key epitopes. Meanwhile, it restrains microglial overactivation and astrogliosis, mitigates neuroinflammation and oxidative stress, and ultimately improves cognitive, learning and memory functions in relevant diseases.

Nevertheless, published studies have presented inconsistent and even conflicting findings, which require critical interpretation. For example, in DSS-induced ulcerative colitis models, some studies reported moderate anti-inflammatory effects of anatabine, whereas others indicated its efficacy was inferior to nicotine. Such discrepancies arise from multiple confounding factors, including differences in animal species and gender, dosage regimens, administration cycles, evaluation indicators and modeling protocols. Similarly, its effects on joint pain and muscle inflammation also show heterogeneity across studies. Some observational studies support obvious symptomatic relief, while controlled trials reveal negligible regulation of TNF-α under exercise-induced muscle injury. These divergent results demonstrate that the pharmacological performance of anatabine depends largely on inflammation subtype, pathological stage and individual physiological status, rather than serving as a universal anti-inflammatory agent.

It is essential to critically evaluate the strengths and inherent limitations of existing preclinical evidence. Current research has clear advantages: well-elucidated molecular mechanisms, consistent phenotypic improvements across various disease models, favorable blood–brain barrier permeability, and superior safety and lower addictive risk compared with nicotine. However, most studies also have prominent methodological flaws. The majority rely on single animal models with small sample sizes, and lack long-term longitudinal observation and systematic dose–response verification. Little attention has been paid to activity differences among stereoisomers, and in-depth mechanistic validation at the cellular and molecular levels remains insufficient. Furthermore, most research stays at the preclinical stage, with few standardized clinical trials. Data regarding pharmacokinetics, long-term toxicity, optimal therapeutic windows and safe dosage ranges are still inadequate.

Although anatabine has shown robust efficacy in a wide range of *in vitro* and preclinical animal studies, substantial gaps remain before its clinical application. Firstly, most experimental data are obtained from rodent and cell models, which cannot fully reflect the complexity of human physiology, disease heterogeneity and individual immune characteristics. Secondly, the lack of systematic pharmacokinetic profiles, long-term toxicological assessment and defined dose-safety relationships creates major barriers to clinical trial design.

In terms of safety concerns, despite its lower addictive potential relative to nicotine, the long-term dependence risk, off-target effects and chronic organ toxicity under prolonged administration have not been fully verified.

Beyond translational challenges and safety risks, multiple bottlenecks also hinder further pharmaceutical development. Low oral bioavailability and rapid metabolic clearance limit its *in vivo* efficacy and long-term administration. In addition, its multi-target characteristics bring therapeutic benefits but obscure the primary target-effect relationship, hindering targeted drug design and structural optimization. The absence of unified experimental standards and patient stratification criteria also makes it difficult to replicate results and form consistent evidence. To bridge the gap between preclinical research and clinical practice, future work should focus on standardized validation across multiple animal species, rational structural modification and novel delivery system development, comprehensive safety and addiction assessment, and well-designed controlled clinical trials, so as to accelerate the clinical translation of anatabine.

Future research directions are summarized as follows. First, conduct standardized multi-model and multi-dose preclinical studies to clarify optimal administration routes, effective dosage ranges and intervention windows, and clarify the causes of conflicting experimental results. Second, further explore stereoisomer activity differences, downstream molecular networks and the crosstalk among nAChRs, NF-κB/STAT3 and Nrf2 pathways to uncover the core pharmacological mechanisms. Third, develop novel dosage forms and delivery systems to enhance bioavailability, brain targeting ability and metabolic stability. Fourth, carry out rigorous long-term toxicological evaluation and controlled clinical trials to confirm the efficacy, safety and addictive liability of long-term medication. Fifth, promote the high-value utilization of waste tobacco resources to build a sustainable industrial development model for anatabine application.

In summary, anatabine is a unique multi-target natural alkaloid with prominent neuroprotective, anti-inflammatory and antioxidant activities, possessing great potential for the treatment of neurodegenerative and chronic inflammatory diseases. Rational interpretation of conflicting data, objective recognition of research limitations and standardized follow-up studies will effectively advance its transformation from laboratory findings to clinical therapeutics.
